# The inhibitory effect of quaternary ammonium salt on bacteria in root canal

**DOI:** 10.1038/s41598-019-48666-4

**Published:** 2019-08-28

**Authors:** Sanjay Kumar Tiwari, Xiao Guo, Yannan Huang, Xuedong Zhou, Hockin H. K. Xu, Biao Ren, Xian Peng, Michael D. Weir, Mingyun Li, Lei Cheng

**Affiliations:** 1 State Key Laboratory of Oral Diseases & National Clinical Research Centre for Oral Diseases, West China Hospital of Stomatology, Sichuan University, Chengdu, 610041 China; 2 Department of Operative Dentistry and Endodontics, West China Hospital of Stomatology, Sichuan University, Chengdu, 610041 China; 3 Department of Endodontics, Periodontics and Prosthodontics, University of Maryland Dental School, Baltimore, MD21201 USA

**Keywords:** Applied microbiology, Biofilms, Pulpitis

## Abstract

Persisting apical periodontitis is a primary reason for multiple intervention in root canal. Persisting bacteria in root canal is related with the persisting infection. Despite the advancement in treatment strategies the persisting infection is a major challenge for endodontist. Here we tested two newly developed quaternary ammonium methacrylates (QAMs) against endodontic bacteria and their biofilms. Their antibacterial and antibiofilm efficiency were compared with chlorhexidine (CHX) and sodium hypochlorite (NaOCl). We measured the MIC, MBC and MBIC of DMADDM and DMAHDM respectively. We also detected the ratio of live/dead bacteria and bacterial composition in the biofilms treated by DMADDM and DMAHDM. We found that DMADDM and DMAHDM could inhibit the growth of bacteria and biofilms formation. The result showed that novel QAMs were remarkably efficient than CHX against biofilms. In addition, we found that *Streptococcus gordonii* (*S*. *gordonii*) and *Enterococcus faecalis* (*E*. *faecalis*) were frequent isolates after treatment with antimicrobial compounds.

## Introduction

Persistent apical periodontitis is a kind of root canal treatment failure, which mainly caused by persistent or secondary microorganisms infection^1^. The presence of microorganisms such as *Enterococcus spp*, nonmutant *Streptococcus spp*, *Actinomyces spp*., and *Lactobacillus spp*, could be detected in the re-infected root canal^2^. It has been reported that *Enterococcus faecalis* (*E*. *faecalis*), *Streptococcus gordonii* (*S*. *gordonii*), *Actinomyces naeslundii* (*A*. *naeslundii*), and *Lactobacillus acidophilus* (*L*. *acidophilus*) were the “core microbiome” because of the high detection rate and stable co-culture station *in vitro*. Among these microbes, *E*. *faecalis* is common isolates from infected root canal.

Chemomechanical intervention intends to bring tooth back to utility by eliminating bacteria from canal. Instrumentation alone is not sufficient for purpose^3^. But, combination with antimicrobial agents produces remarkable results^4^. Nevertheless, bacteria survive in prepared canal unfavorably impact outcome^5–7^. Various irrigation strategies are developed to combat with persisting bacteria in canal^4^. Sodium hypochlorite (NaOCl) is first choice as an irrigation because it could kill bacteria and disrupt biofilm^4^. Since, NaOCl is corrosive to dentine chlorhexidine is an alternate choice and its antimicrobial spectrum is comparable to NaOCl^8,9^. But, CHX lacks the ability to disrupt the biofilm that protects bacteria present in depth of biofilm from antimicrobial agents^10^. CHX treatment fixes the biofilms to dentine wall which makes it necessary for use of other compound that will disrupt the biofilms and chlorhexidine will kill bacteria^11^. New antimicrobial compound is needed that could kill bacteria and disrupt biofilms so that clean dentine surface is obtained and dental sealers can bind to dentine.

Recently, two quaternary ammonium methacrylate’s (QAMs) compound, dimethylaminododecyl methacrylate (DMADDM), and dimethylaminohexadecyl methacrylate (DMAHDM) are developed. When they were incorporated in different dental materials, they showed lasting and remarkable antibacterial effect and good biocompatibility. Besides, it’s reported that DMADDM incorporated in dental materials had the ability to alter the biofilm structure to a healthier condition^12,13^. But, the efficiency of novel QAMs monomers to eradicate the endodontic biofilm is not yet defined.

The aim of this study was to determine whether new QAMs compounds could eradicate endodontic bacteria and disrupt their biofilms. The capacity of antimicrobial compounds will be tested by Live/Dead ratio, analysis the composition of bacteria in the biofilm and observation the structure of biofilm. *S*. *gordonii*, *E*. *faecalis*, *L*. *acidophilus* and *A*. *naeslundii* were four endodontic bacteria selected to confirm our hypothesis. We hypothesized that DMADDM and DMAHDM could inhibit the growth of bacteria and the formation of biofilms.

## Results

### MIC, MBC, and MBIC of DMADDM and DMAHDM

MIC, MBC, and MBIC of DMADDM against combined four endodontic bacteria were 50 µg/mL, 100 µg/mL, 25 µg/mL, respectively. The MIC, MBC and MBIC of DMAHDM for the four bacteria are 12.5 µg/mL, 12.5 µg/mL, 6.25 µg/mL, respectively. The MBIC was half of MIC for both of DMADDM and DMAHDM. MBC of DMAHDM did not change but MBC of DMADDM was twice of its MIC.

### DMADDM and DMAHDM inhibited the growth of bacteria and biofilms formation

**Figure 1:**
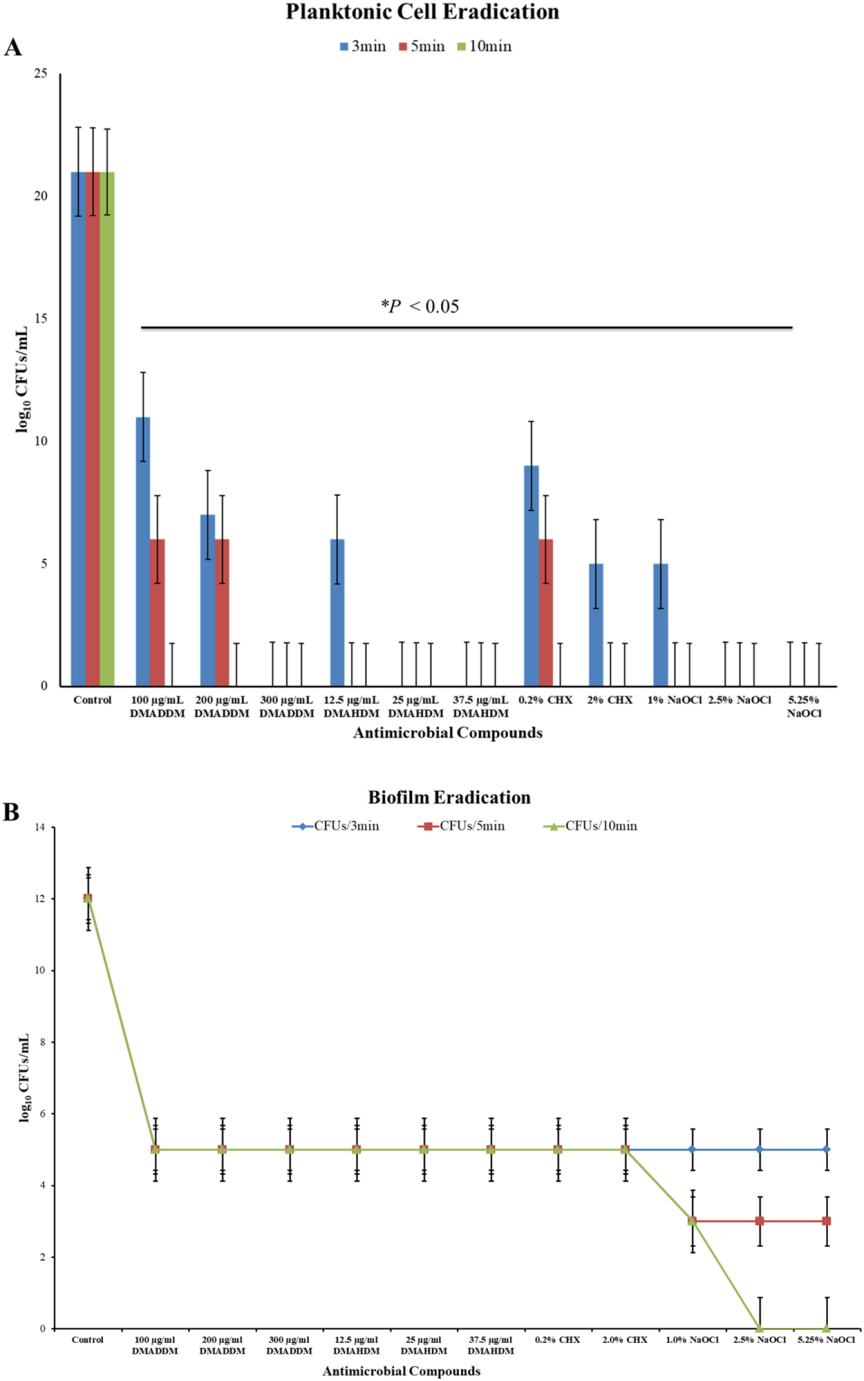
(**A**) Planktonic bacteria eradication analysis by DMADDM, DMAHDM, CHX and NaOCl at different concentrations for 3 min, 5 min and 10 min. Each value is mean ± standard deviation (n = 9) *P < 0.05. (**B**) log CFUs of bacteria after treatment with different concentrations of DMADDM, DMAHDM, CHX and NaOCl for 3 min, 5 min and 10 min. Each value is mean ± standard deviation.

Compared to control group, all antimicrobial compounds significantly reduced CFUs from planktonic suspension and biofilms (*P* < 0.05) (Fig. 1). All planktonic bacteria (Fig. 1A) were killed by 100 µg/mL DMADDM in 10 min, and 300 µg/mL DMADDM in 3 min. For the DMAHDM, all planktonic bacteria were killed by 12.5 µg/mL in 5 min, and 25 µg/mL in 3 min.

In 3 min, all compounds reduced colony counts by 7 logs from biofilms and after wards remained constant during remaining two observations (Fig. 1B). 5.25% NaOCl eliminated most cells from biofilm at 10 min (Fig. 1B).

**Figure 2:**
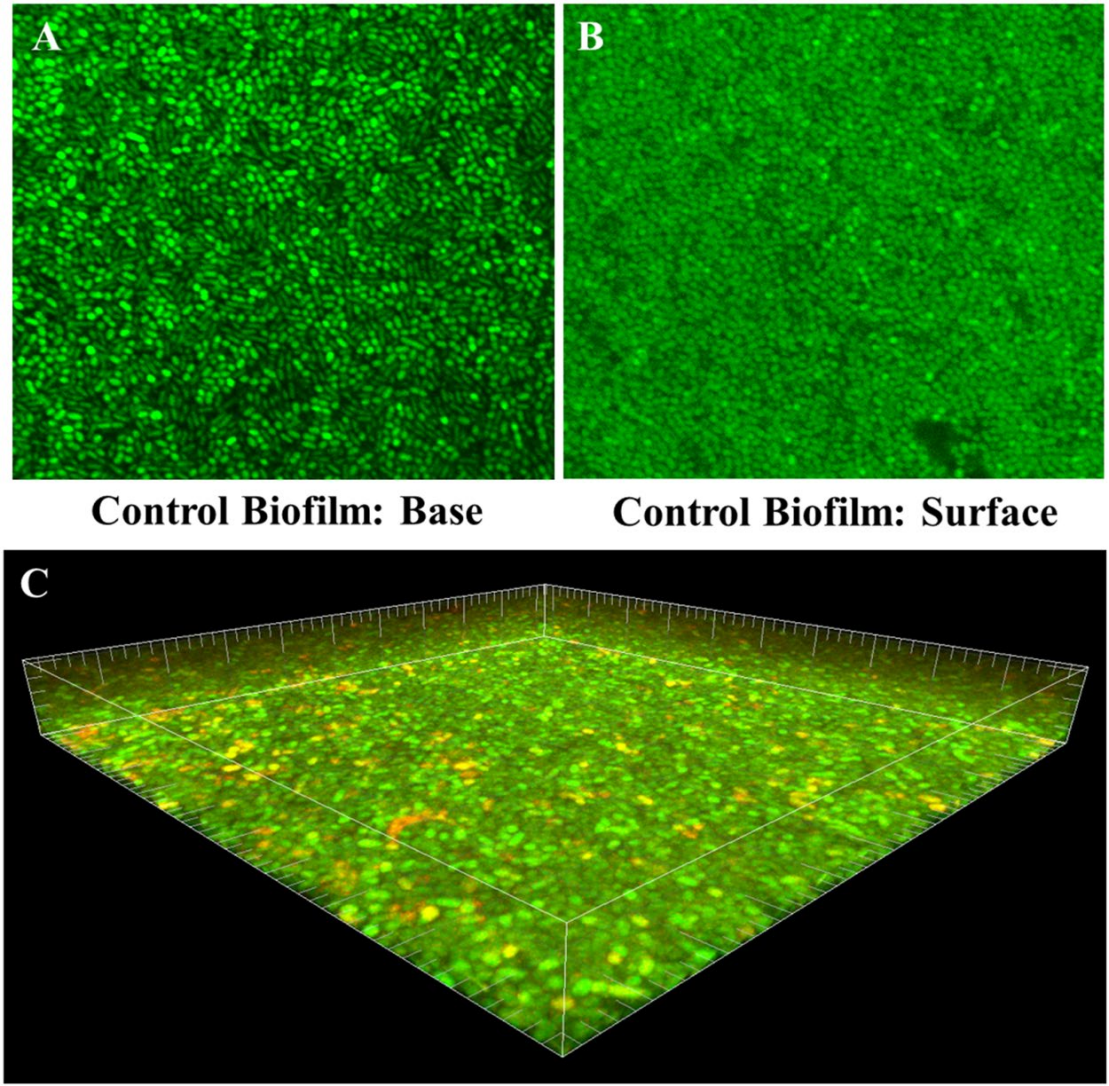
CLSM image of control biofilm. (**A**) Image of base of Biofilm, it shows base of biofilm dominated by rod shaped bacteria. (**B**) Surface of biofilm shows the dominance of coccid shaped bacteria. (**C**) 3D image of control biofilm.

**Figure 3:**
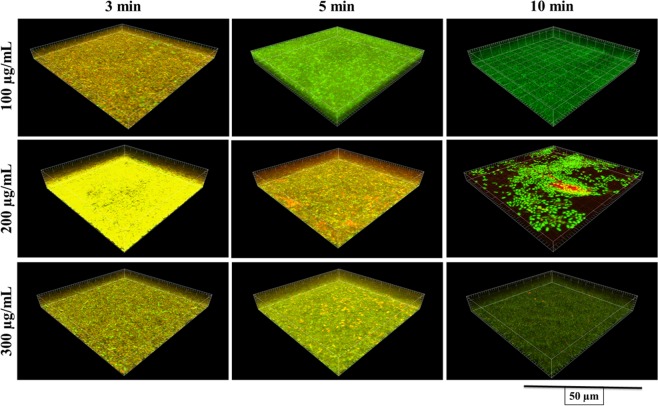
CLSM image of 48 h biofilms after treatment with different concentrations of DMADDM for 3 min, 5 min and 10 min.

**Figure 4:**
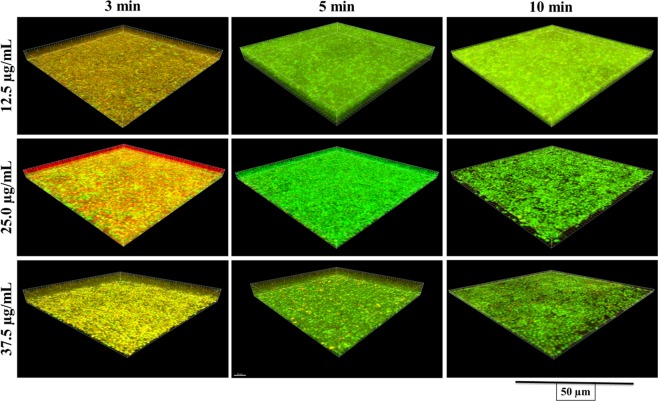
CLSM image of 48 h biofilms after treatment with different concentrations of DMAHDM for 3 min, 5 min and 10 min.

**Figure 5:**
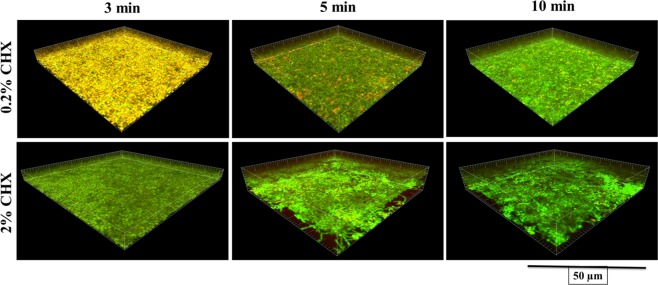
CLSM image of 48 h biofilms after treatment with different concentrations of CHX for 3 min, 5 min and 10 min.

**Figure 6:**
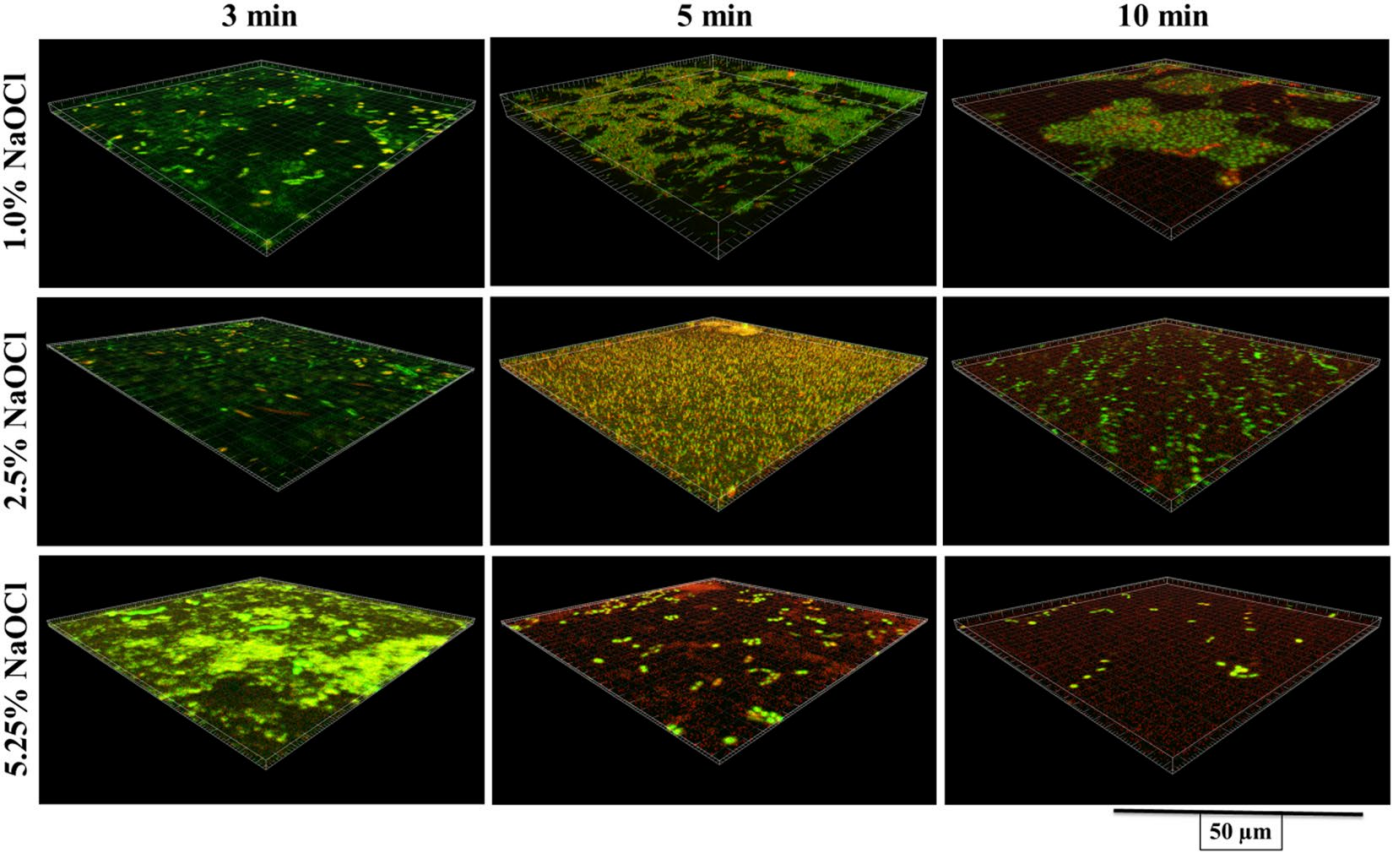
CLSM image of 48 h biofilms after treatment with different concentrations of NaOCl for 3 min, 5 min and 10 min.

**Figure 7:**
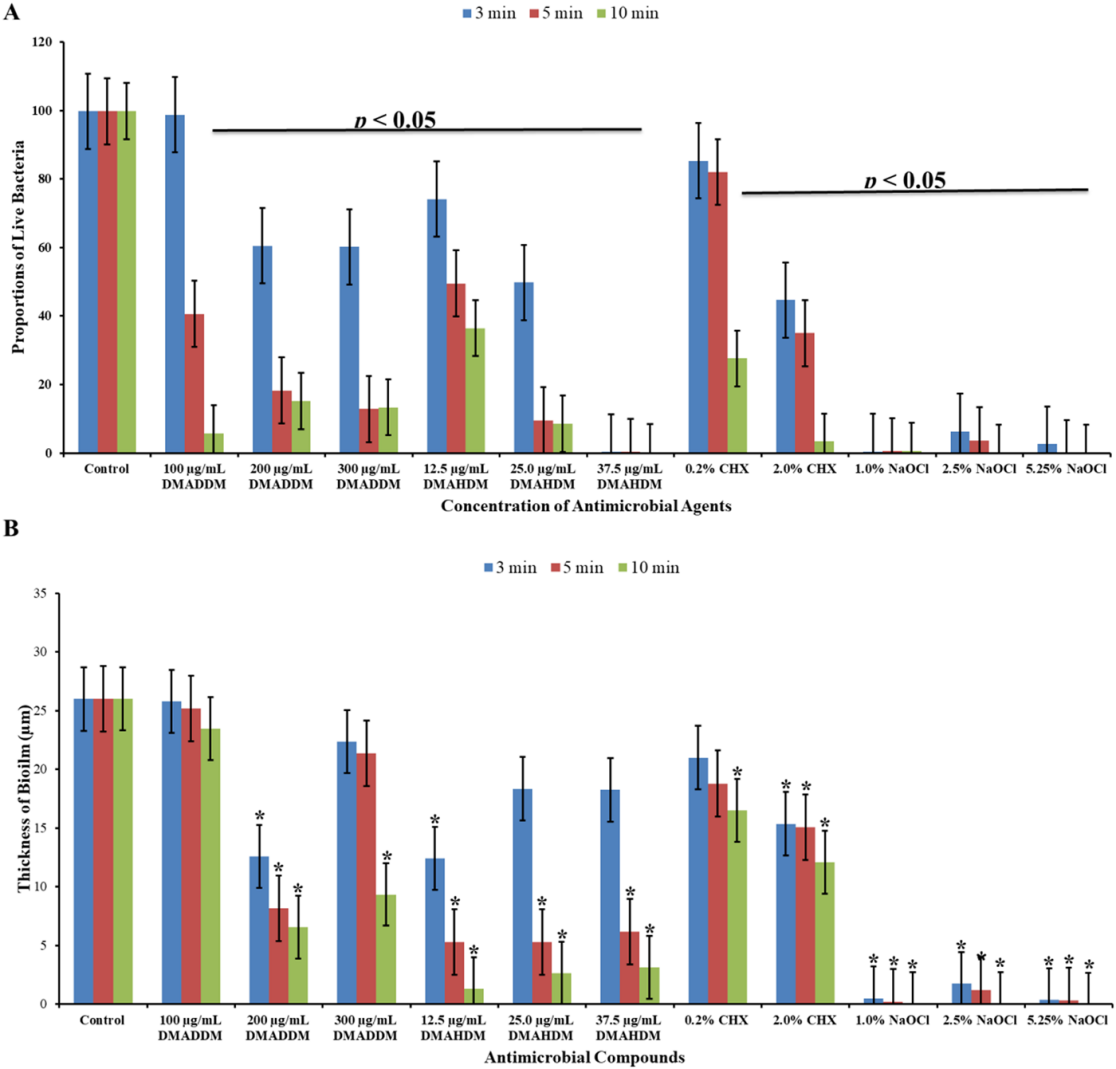
(**A**) Proportion of live bacteria in biofilm after treatment with DMADDM, DMAHDM, CHX and NaOCl for 3 min, 5 min and 10 min. (**B**) Thickness of biofilm after treatment with antimicrobial compounds. Each group name and contact duration is indicated in Image. Higher concentrations of DMADDM and DMAHDM, and increased duration of contact with biofilm provided stronger antimicrobial effect. Data is presented in mean ± standard deviation. *P < 0.05.

Figure 2 shows live and dead bacteria in control group. Live bacteria were stained green and dead bacteria were stained red. Yellow/orange color stains were seen more in biofilms treated for 3 min, except NaOCl. Figures 3–7 showed thickness of biofilm were reduced after treatment. 200 µg/mL DMADDM and 12.5 µg/mL DMAHDM significantly inhibited biofilms (*P* < 0.05) (Figs 3 and 4). DMADDM and DMAHDM significantly disrupted much more biofilms than CHX (*P* < 0.05) and the effect of disruption was similar to NaOCl. Increased concentrations of compounds and increasing contact time significantly killed more bacteria and left less live cells in biofilms (*P* < 0.05) (Fig. 7).

### The composition of biofilms bacteria treated by antibacterial compounds

**Figure 8:**
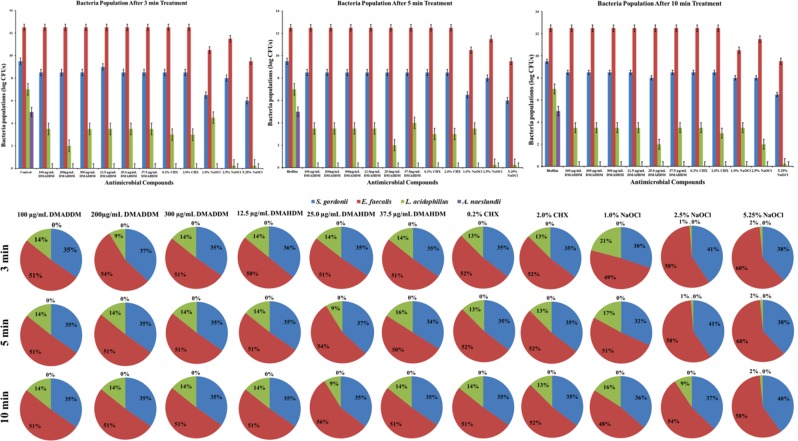
The compositions shift of *A*. *naeslundii*, *L*. *acidophilus*, *S*. *gordonii* and *E*. *faecalis* in biofilms after treatment with DMADDM, DMAHDM, CHX and NaOCl for 3 min, 5 min and 10 min. The composition shift was determined by TaqMan real-time polymerase chain reaction. No significant differences in proportions shifts were observed among groups.

Composition of four bacteria in biofilms after treatment with DMADDM, DMAHDM, CHX and NaOCl for 3 min, 5 min and 10 min are shown in Fig. 8. Changes in proportion of bacteria are represented in bar graph and pi-chart. There was no significant difference in recoveries of bacteria from biofilm after treatment between specimens (*P* > 0.05). But contradictory results were found when the treated samples were incubated anaerobically on agar plate, *S*. *gordonii* and *E*. *faecalis* were the only bacteria growing and result was confirmed from qPCR (data not shown).

## Discussion

The present study investigated the antibacterial and antibiofilm property of DMADDM and DMAHDM against CHX and NaOCl in multispecies biofilm for the first time. The results indicated that DMADDM and DMAHDM could significantly kill bacteria and disrupt biofilms in endodontic bacteria (*P* < 0.05). These compounds not only possessed great antibiofilm effect, but also could inhibit bacteria to form the biofilms. This is favorable for prevention of recurrent infection due to re-growth residual bacteria in root canal. This study used DMADDM and DMAHDM as endodontic irrigant to investigate its antibacterial effect on controlled multispecies biofilms grow *in vitro*.

The study was focused on eradication of biofilm which is safe harbor for bacteria and hub for antimicrobial resistance code transfer between bacteria^14^. There are growing evidences that show presence of antibiotic resistant bacteria and resistant genes in endodontic biofilm^14–16^. Nowadays, world is under crisis of antibiotic and threat of emerging resistant bacteria against available antibiotics. Thus, it is necessary to eliminate biofilms from root canals and stop spread of resistance.

Firstly, endodontic infection is caused of multispecies and experiment designed for endodontic purpose should mimic the clinical situation. *Streptococcus* spp., *Enterococcus* spp., *Lactobacillus* spp., and *Actinomyces* spp. are common isolates from primary infection and persisting periapical lesion cases^16^. Secondly, multispecies specimens show increasing resistance against antimicrobial compounds than mono-species specimen^17,18^. So, we selected multispecies specimen for our study.

*A*. *naeslundii*, *E*. *faecalis*, *L*. *acidophilus and S*. *gordonii* play unique roles in biofilms. In presence of *A*. *naeslundii*, *S*. *gordonii* forms stable coaggregation partnership, develops strong intergeneric communications, colonizes better than in mono-species biofilm^19^, and *A*. *naeslundii* could protect *S*. *gordonii* from self-produced hydrogen peroxide (H_2_O_2_)^20,21^. *E*. *faecalis* common isolated from root canal with persistent apical periodontitis^22^, is capable to survive in harsh environment increasing the survival of other bacteria in biofilms and enhancing the virulence of biofilms^22–24^.

Treatment significantly (*P* < 0.05) reduced CFUs from both planktonic suspension and biofilms (Fig. 1). Most planktonic bacteria got eliminated within 5 min of treatment but bacteria in biofilms still grow on agar plate after 10 min of treatment. If bacteria cannot be eliminated from biofilms, it is necessary to inhibit bacteria form biofilms. We found that ½ MIC (i.e. MBIC) of DMADDM and DMAHDM could inhibit bacteria form biofilms effectively. Thus, irrigation of root canal with DMADDM and DMAHDM at MBIC concentration before obturation will be sufficient to inhibit biofilm formation. Biofilm formation is guided by gene and it’s not known whether DMADDM and DMAHDM inhibited gene expression or only made bacteria incapable to form biofilms.

NaOCl is first choice antimicrobial compound for canal irrigation^25^. 5.25% NaOCl killed bacteria and disrupted biofilm (Figs 1, 6 and 7). But it is corrosive to exposed dentine thus not suitable as final irrigant^8,26^. CHX is an alternate choice for final irrigation^4,27^ and our data also shows that 2.0% CHX owns better antibacterial and antibiofilm effect. When the DMADDM and DMAHDM are compared with CHX or NaOCl, at lower concentration (1% = 10,000 µg/mL) the novel compounds were equally effective as CHX and NaOCl in killing bacteria and disrupting biofilm. So, DMADDM and DMAHDM could be better alternative for irrigation in future.

CHX binds to the negatively charged bacteria cell wall and causes cell membrane disruption^9^. QAMs are cationic compounds and kills bacteria by cell wall disruption^28^. It was seen that bacteria resistance against CHX showed cross resistance to DMADDM. It might be CHX and DMADDM sharing common mechanism for cell lysis. It is believed that QAMs form free volume in cell membrane which brings imbalance in membrane integrity and cell disrupts under its own intracellular pressure. The free volume formation is dependent on QAMs chain length and DMAHDM forms optimum free volume^29^. From above analysis, it could be assumed that DMADDM and CHX either could not form free layer adequately or they are pumped out from cell before they could get incorporated in cell membrane^17,29^. But, the reasons for cross resistance between DMADDM and CHX are not yet understood.

The study of live and dead cells showed that biofilms treated for 3 min have more yellow/orange fluorescence rather than definite green or red fluorescence. This also indicated that cells are dead^30^. High proportion of dead bacteria in biofilms could be due to bacteria programmed death after receiving stress signals released from bacteria on surface of biofilms. The data for CFUs count indicated that there were no changes in colony counts among 3 min, 5 min and 10 min treatment with CHX and QAMs, while CLSM analysis showed more dead cells. It could be programmed cell death or diffusion of compound inside biofilms or it was the remaining effect by QAMs and CHX because biofilms were not treated with neutralizing compound after treatment and before staining.

This study identified antimicrobial and antibiofilm efficiency of DMADDM and DMAHDM against endodontic bacteria. DMADDM and DMAHDM performed better affection than CHX against biofilms. NaOCl disrupts biofilms, kills bacteria and dissolves remnant formed by dead bacteria on surface of biofilms^31^. DMADDM, DMAHDM and CHX are lack of the ability to dissolve remnant. But, remnant did not interfered DMADDM and DMAHDM to kill bacteria from deep inside biofilms and further disrupt biofilms (*P* < 0.05). Biofilm disruption is an inherent property of NaOCl, 2.0% CHX disrupt biofilms in some extent, but DMADDM and DMAHDM have remarkable effect. These analyses show the potential future of DMADDM and DMAHDM as an endodontic irrigant.

Except, *A*. *naeslundii* qPCR result showed no changes in bacteria proportions from all cases after treatment. This could be either *S*. *gordonii*, *L*. *acidophilus* and *E*. *faecalis* are more resistant towards antimicrobial treatment or the DNA of death cells remained trapped in biofilm matrix and got expressed during PCR cycle. So, to eliminate our doubt from qPCR analysis of biofilm, we incubate biofilm samples to grow in BHI broth after treatment and its qPCR analysis showed *S*. *gordonii* and *E*. *faecalis* grow in broth but *L*. *acidophilus* were not detected. This confirms that these two bacteria are resistant to antimicrobial agents (Fig. 8).

We further extended our work to analyze the interactions between bacteria during biofilm formation (data shown in supplementary materials). When samples from three bacteria species biofilms were spread on agar plates, *E*. *faecalis* and *S*. *gordonii* were only two bacteria to grow. This showed that *S*. *gordonii* and *E*. *faecalis* together could survive in multispecies biofilms and *L*. *acidophilus* got inhibited in three species biofilms. The mechanism of survival of these two bacteria together is unknown but it is confirmed that they could survive together after treatment of antimicrobial compounds. Further analysis of *E*. *faecalis* and *S*. *gordonii* showed that they together formed biofilms with less biomass and thinner biofilms, images from CLSM showed cells with intact cell wall and more live cells, but few *S*. *gordonii* were recovered on agar plate than *S*. *gordonii* from mono-species biofilms. But there was no significant change in colony numbers from *E*. *faecalis* when grown mono-species or dual-species biofilms.

SEM image (Fig. S2) of control group showed that *E*. *faecalis* in vicinity with *L*. *acidophilus* have damaged cell wall and Gao *et al*. had found that *L*. *acidophilus* could inhibit *E*. *faecalis*^24^. In order to justify these findings, we formed two biofilms samples, first samples contained both bacteria in equal proportions (i.e. at 0.2 OD_600 nm_) and second biofilm was formed with *L*. *acidophilus* and *E*. *faecalis* at 0.4 OD_600 nm_ and 0.2 OD_600 nm_, respectively, to see if *L*. *acidophilus* in higher quantity will completely eradicate *E*. *faecalis*. We found that biofilms formed with both bacteria in equal OD_600 nm_ had more dead *E*. *faecalis* than double *L*. *acidophilus* (Fig. 4B, S2B–D, S2D). This showed that *L*. *acidophilus* could inhibit *E*. *faecalis*, but could not eliminate *E*. *faecalis* completely.

*Lactobacillus* spp. is commonly associated with dental caries. But these bacteria were found to have inhibitory action against *Streptococcus mutans*^32,33^ which is major cause for dental caries. Similarly, *Lactobacillus* spp. supplement has shown to have positive outcome after treatment^34–38^. This is an encouraging finding, and further investigation is needed to identify the mechanism adopted by *Lactobacillus* spp. to inhibit other bacteria. The results from further work could be used to control *E*. *faecalis* and other pathogens that are commonly associated with persisting apical periodontitis.

In conclusion, we could say that novel QAMs antimicrobial compounds could inhibit bacteria growth and biofilms formation. Among, the hypothesis designed for our QAMs, they proved to be equally effective against endodontic bacteria and endodontic biofilm. Thus, they have potential to be used as an endodontic irrigant in future. The interactions between bacteria also plays key role during biofilms formation, we also need more tests to verify our findings.

## Materials and Methods

### Synthesis of QAMs and preparation of working solutions

DMADDM and DMAHDM were produced as described previously^39^. The 5 mg/mL stock solutions of both compounds were prepared in sterilized phosphate buffered saline (PBS). The working concentration of DMADDM and DMAHDM were prepared from stock solution.

5.25% NaOCl and 20% CHX (Sigma, China) solutions were purchased from suppliers and were diluted before uses.

### Bacteria inoculation and Biofilm formation

*S*. *gordonii (*ATCC 10558), *E*. *faecalis* (ATCC 19433), *L*. *acidophilus* (ATCC 4356), and *A*. *naeslundii* (ATCC 12104) were provided by State Key Laboratory of Oral Diseases (Sichuan University, China) and were routinely cultured anaerobically (90% N_2_, 5% CO_2_, 5% H_2_) in Brain-heart infusion (BHI; Difco, Sparks, MD, USA) broth at 37 °C. Equal volumes of bacterial suspension at optical density (OD) 0.2 were combined to form mixed bacteria suspension for this experiment.

For the biofilm formation, bacterial suspensions 0.2 (OD_600 nm_) were mixed to obtain an inoculum containing equal volumes of *S*. *gordonii*, *E*. *faecalis*, *L*. *acidophilus*, and *A*. *naeslundii* in 2 mL of BHI with 1% sucrose in 24-well plates and incubated anaerobically for 48 h at 37 °C. Biofilms for disinfection analysis was grown in 200 µL in 96-well plate.

### Determining MIC, MBC and MBIC of DMADDM and DMAHDM

Minimum inhibitory concentration (MIC) was concluded by broth microdilution in 96-well microtiter plate (Corning Incorporated, NY, USA) as described previously^17^. Plates were incubated anaerobically at 37 °C for 24 hours. MIC was defined as the lowest concentration of antimicrobial agent that inhibit visible bacterial growth and minimum bactericidal concentration (MBC) was the lowest concentration of antimicrobial compound that reduced the inoculant by 3 logs (i.e. no more than 1 to 2 colonies on agar plate)^17^.

100 µL of BHI culture medium was added to each well in 96-well plates, then 200 μg/mL DMADDM and 100 μg/mL DMAHDM were added to the first well and serial dilutions were made with 100 μL of BHI liquid medium to obtain a series of concentration gradients. After incubation at 37 °C anaerobically for 48 h, the lowest concentration at which there was no visible bacterial growth was recorded as the MIC value.

The minimum biofilm inhibition concentrations (MBICs) were identified by allowing bacteria to form biofilm in presence of DMADDM and DMAHDM in 96-well plate at 37 °C anaerobically. The selection of QAMs concentrations for MBIC was similar to MIC concentrations. MBIC was determined as described previously^40^. Briefly, 200 μL of the bacterial suspension was added to 96-well plate, and cultured for 4 hours anaerobically at 37 °C to make bacteria adhered to the bottom. After the bacteria adhered, the biofilm was rinsed once to wash away the loosely bacteria. Then 200 μL of BHI containing 200 μg/mL DMADDM and 100 μg/mL DMAHDM was added and the plate was cultured anaerobically at 37 °C for 48 hours to form biofilms. After the biofilm formation, the biofilms on the bottom of the microplates washed twice with PBS. Then the biofilms were stained with 200 µL of 0.5% (w/v) crystal violet for 10 min and washed by PBS there times to remove the residual dye. 200 μL of absolute ethanol was added to release the bounded dye. 100 μL of the solution was taken from each well and transferred to a new 96-well plate, and the absorbance was measured at a light wavelength of 600 nm (A600 nm) using a microplate reader (EON, BioTek, USA).

### Disinfection of Planktonic bacteria

Mixed suspension of Planktonic bacteria in PBS at 0.2 (OD_600 nm_) was exposed to the different concentrations of DMADDM, DMAHDM, NaOCl and CHX for 3, 5 and 10 min. The procedure for planktonic bacteria disinfection was adopted from previous study^41^ with some modifications. We took 1xMBC, 2xMBC and 3xMBC concentrations of DMADDM and DMAHDM in this study. After treatment, 10 µL of suspension were plated on BHI agar plate after serial dilutions and colony forming units (CFUs) were counted after 48 h of an anaerobic incubation at 37 °C.

### Analysis the biofilm formation from mixed bacteria culture

After biofilm formation for 48 h, biofilms were washed twice with PBS and treated with DMADDM, DMAHDM, NaOCl and CHX for 3, 5 and 10 min.

Biofilms were stained using Baclight live/dead viability dyes (Molecular Probes, Eugene, OR, USA). Briefly, the LIVE and DEAD dye was prepared by mixing SYTO 9 and Propidium iodine in ultrapure water at proportion of 1:100. 50 µL of dye was added in center of biofilm and left in dark at room temperature for 15 min. The biofilms were observed randomly at least 5 different under confocal laser scanning microscope (CLSM, BX51, Olympus, Tokyo, Japan) with 60x oil immersion objective lens^42^. The three-dimensional reconstruction of biofilms was performed with IMARIS 7.0.

For qPCR analysis, DNA was extracted using TIANamp Bacteria DNA kit (TIANGEN, Beijing, China) following manufacturer’s guideline. Enzymatic lysis of Bacteria cell were done with lysis buffer (20 mM Tris-HCL, pH 8.0; 2 mM sodium EDTA and 1.2% Triton X-100) containing 20 mg/mL lysozyme for 1.5 h at 37 °C. Pureness and quantity of extracted DNA was examined in NanoDrop 2000 spectrophotometer (Thermo Scientific, Waltham, MA, USA). TaqMan real-time polymerase chain reaction (Life Technologies, Carlsbad, CA, USA) was done to identify the absolute number of *A*. *naeslundii*, *E*. *faecalis*, *L*. *acidophilus* and *S*. *gordonii* in biofilm. The sequences of probes were labeled in Table 1.

**Table 1:** The species specific primers used in qPCR to identify prevalence of individual bacteria in biofilm.

Bacteria	Primer pairs
*E*. *faecalis*	F: 5′-CGCGAACATTTGATGTGGCT-3′
	R: 5′-GTTGATCCGTCCGCTTGGTA-3′
*S*. *gordonii*	F: 5′-GCCTTAATAGCACCGCCACT-3′
	R: 5′-ÇCATCTCTGTTGTTAGGGCGT-3′
*L*. *acidophilus*	F: 5′-AGAGGTAGTAACTGGCCTTTA-3′
	R: 5′-GCGGAAACCTCCCAACA-3′
*A*. *naeslundii*	F: 5′-CTCCTACGGGAGGCAGCAG-3′
	R: 5′-CACCCACAAACGAGGCAG-3′

### Analysis

Each experiment was repeated in triplicate using independent bacteria cultures. Data were analyzed by one-way analysis of variance (ANOVA). The differences were reported at a significance level of 0.05.

The CLSM images were analyzed with bioImageL software^42^.

## Supplementary information

Dataset
