# Supplementary material for: The inhibitory effect of quaternary ammonium salt on bacteria in root canal
Source: Sci Rep. 2019 Aug 28;9:12463. doi: 10.1038/s41598-019-48666-4 (PMC6713705; doi:10.1038/s41598-019-48666-4)
Supplement: Supplementary file 1 — Dataset [file 41598_2019_48666_MOESM1_ESM.docx]

**The inhibitory effect of quaternary ammonium salt on bacteria in root canal**

Sanjay Kumar Tiwari^1,2#^，Xiao Guo^1,2#^, Yannan Huang^1,2#^, Xuedong Zhou^1,2^, Huakun (Hockin) Xu ^3^, Biao Ren^1^, Xian Peng^1^, Michael D. Weir^3^, Mingyun Li^1*^, Lei Cheng^1,2*^

**Appendix**

Crystal Violet Staining Assay

Biofilms were rinsed by PBS twice, then fixed by 100% methyl alcohol for 15 min. The biofilms on the bottom of the microplates were stained with 200 µL of 0.1% crystal violet for 5 min and then washed with sterile distilled water to remove the residual dye. The bounded crystal violet was released by 200 µL 95% ethanol. The absorbance of released crystal violet in ethanol was recorded at OD_600 nm_ by a spectrophotometer.

Biofilms structure observation by a scanning electron microscope (SEM)

After culturing in BHI medium, biofilms were washed with PBS to get rid of loosely bacteria adhering bacteria on before fixing in 1% glutaraldehyde for 4 hours at 4ºC. Specimens were washed with PBS and subjected for dehydration in increasing ethanol concentrations (30%, 50%, 70%, 80%, 85%, 90%, 95%, and 100%) for 15 min. Finally, the biofilms were gold sputter-coated and inspected under SEM (INCA Penta FETX3, OXFORD INSTRUMENT)^1^**.**


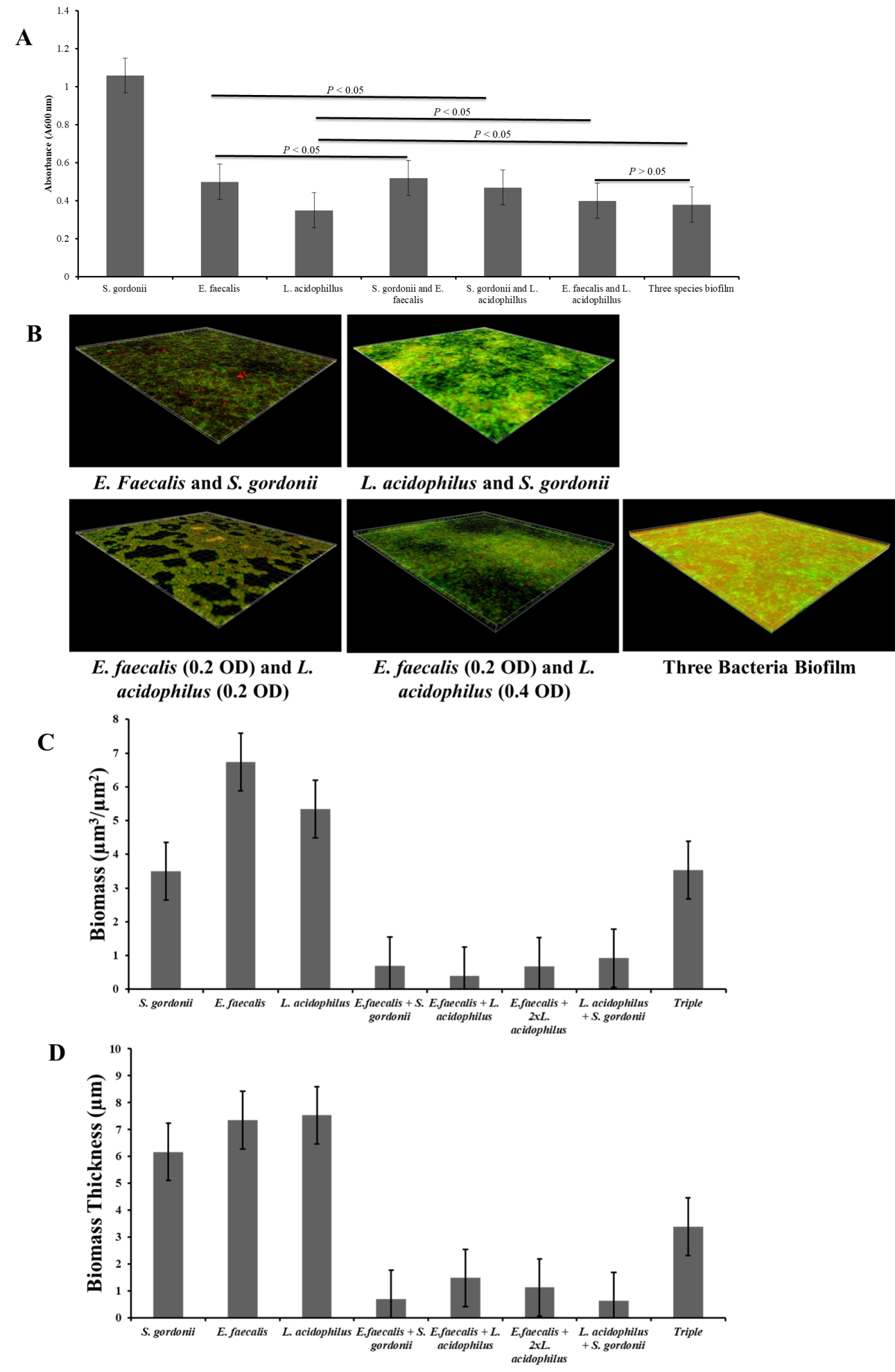


**Figure S1.** The study of dual and three species biofilms. (A) Absorbance reading from quantification of biofilms with crystal violet (OD_600 nm_). (B) CLSM and SEM image of dual and three species biofilm (live bacteria, green stain; dead bacteria, red stain). (C) Analysis of biomass volume of biofilm from CLSM image. (D) Analysis of biomass thickness of biofilm from CLSM image. **P* < 0.05.


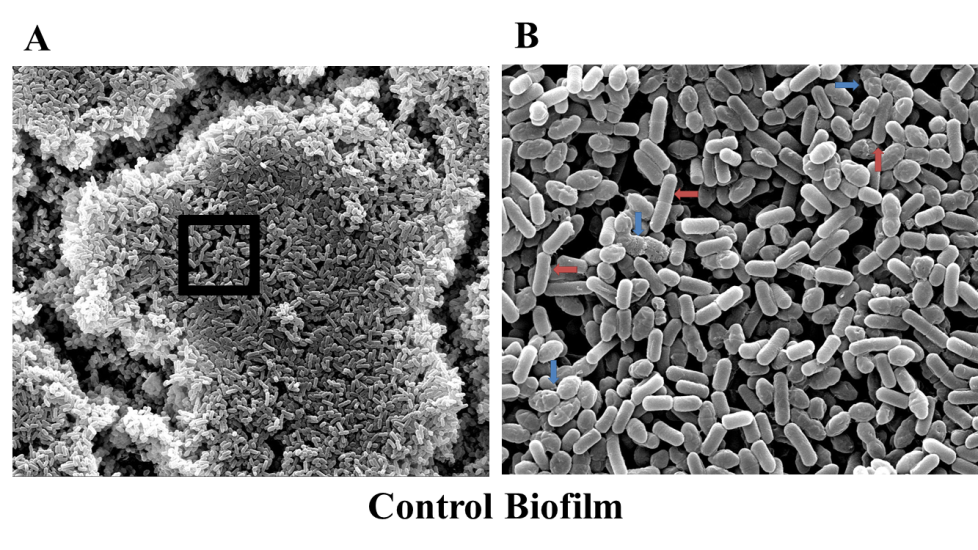


**Figure S2.** Scanning electron microscopy image of multispecies biofilms. **(A)** SEM image of control biofilm formed by *A. naeslundii, E. faecalis, L. acidophilus* and *S. gordonii.* **(B)** Enlarged image of control biofilm*.*

1 Chavez de Paz, L. E. Image analysis software based on color segmentation for characterization of viability and physiological activity of biofilms. *Appl. Environ. Microbiol.* **75**, 1734-1739, doi:10.1128/AEM.02000-08 (2009).
